# Juxta-adrenal schwannoma presenting as a giant adrenal tumor: A case report and a literature review

**DOI:** 10.1016/j.ijscr.2018.10.017

**Published:** 2018-10-15

**Authors:** Maher Abdessater, Mohammad El Mokdad, Jerome Gas, Walid Sleiman, Patrick Coloby, Stephane Bart

**Affiliations:** Centre Hospitalier Régional René DUBOS, Pontoise, France

**Keywords:** Retroperitoneal schwannoma, Adrenal tumor, Misdiagnosis, Case report

## Abstract

**Introduction:**

Retroperitoneal schwannomas (RS) are rare, benign tumors that originate in the neural sheath. Juxta-adrenal schwannomas may be misdiagnosed with giant adrenal tumors. This article reports the case of a RS that presented as an asymptomatic adrenal mass in a 50 Y.O female.

**Presentation of case:**

An abdominal ultrasound of our asymptomatic patient showed right adrenal lesion of 9 cm of diameter. Endocrinological evaluation was negative. The patient was considered to have a non-secreting right adrenal mass confirmed by adrenal scan. We began a right laparoscopic *trans* peritoneal adrenalectomy, but when we discovered intra operatively that the wall of the IVC and the renal vein were very adherent to the mass which had a lot of small vessels that were bleeding, we converted to open surgery that allowed us to remove the mass safely. The operative time was 200 min, the blood loss was 850 cc and the patient was discharged uneventfully on the sixth day after surgery.

**Discussion:**

Although we thought that we removed a huge adrenal tumor from the retroperitoneum of our patient, the pathological exam revealed a RS that comprises the adrenal gland which was normal. Preoperative establishment of diagnosis is difficult in case of RS that can be misdiagnosed, especially when they stick to other structures (the adrenal in our case).

**Conclusion:**

Complete surgical resection is the treatment of choice for RS and open surgery is the safest option when we have big tumors. Histology and Immunohistochemistry confirms the diagnosis that can be easily missed preoperatively.

## Highlights

• Retroperitoneal schwannomas (RS) are rare, benign tumors that originate in the neural sheath.
• RS can be misdiagnosed preoperatively, especially when they stick to other structures (the adrenal in our case).
• Complete surgical resection is the treatment of choice and open surgery is the safest option when we have big tumors.
• Histology and Immunohistochemistry confirm the diagnosis.

## 1 Introduction

Retroperitoneal masses are of large varieties ranging between rare benign tumors and malignant neoplasms that can be either primary or metastatic [1]. Schwannomas comprise almost 1% of these masses [2]. The large and flexible retroperitoneal space gives the time to the tumor to expand, causing no symptoms until reaching other retroperitoneal organs (as the adrenal gland), this is why the diagnosis is often delayed and the lesion can reach a significant and late stage at the time of diagnosis [3]. Juxta-adrenal schwannomas may be misdiagnosed with giant adrenal tumors, and only a few cases have been reported in the literature [4,5]. In this article, which has been reported in line with the SCARE criteria [6], we report the case of a retroperitoneal schwannoma that presented as an asymptomatic adrenal mass in a 50 year old female patient.

## 2 Case report

**Fig. 1:**
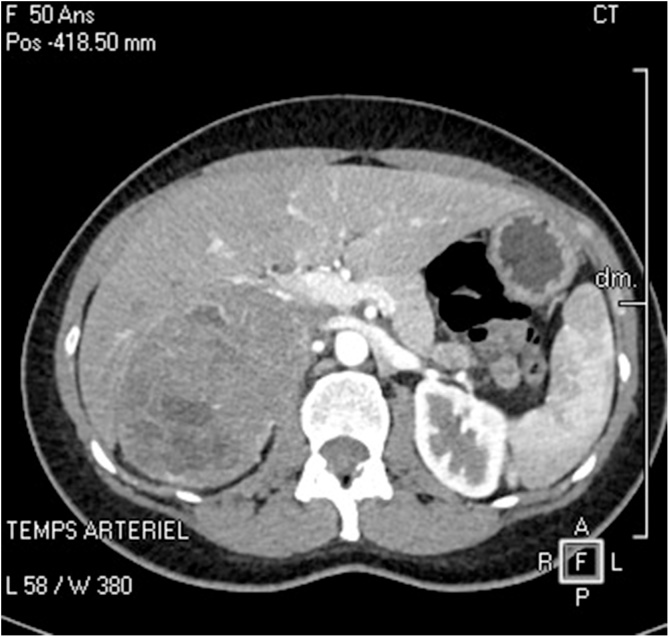
Adrenal scan showing a well encapsulated 10 × 9 × 7 cm heterogeneous right adrenal mass with areas of necrosis and calcifications without local invasion.

**Fig. 2:**
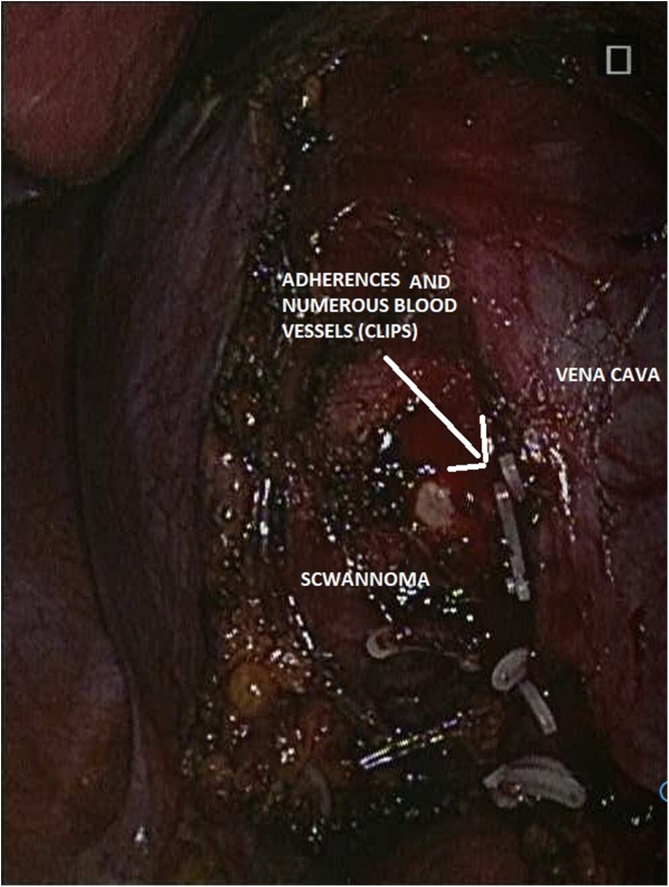
Laparoscopic view showing adherences between the wall of the IVC and the mass which had a lot of small vessels that were clipped.

**Fig. 3:**
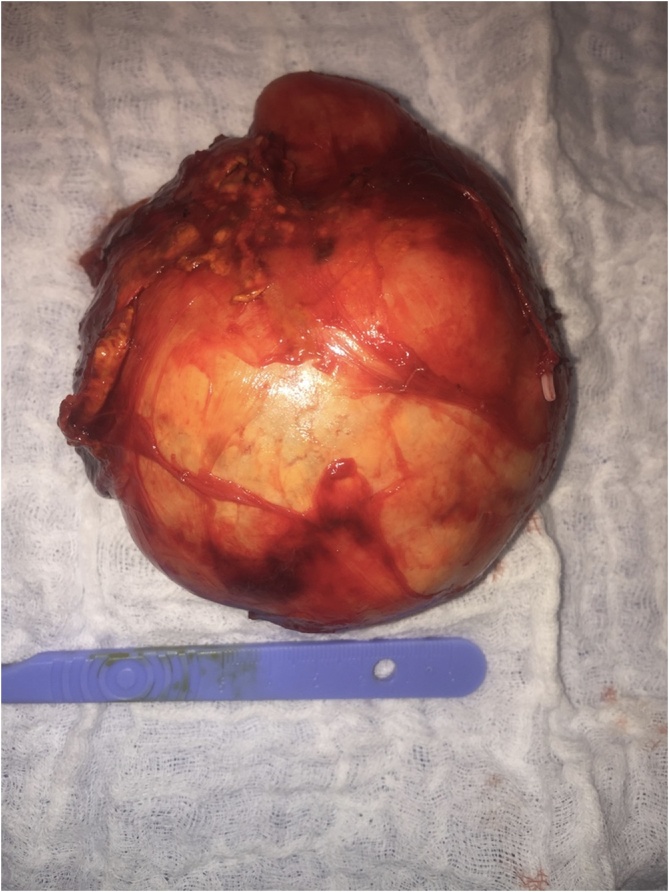
Macroscopic aspect of the tumor before opening it.

A 50 year old female patient previously healthy has undergone an abdominal ultrasound demanded by her primary care physician when her routine checkup blood test showed a slightly elevated level of liver enzymes with no other lab abnormalities. This ultrasound showed right adrenal lesion of 9 cm of diameter. An Abdominal MRI was then done and revealed a soft tissue necrotic encapsulated mass of 10 × 9 cm of right adrenal gland origin. She was completely asymptomatic and the physical exam was strictly normal. Endocrinological evaluation was done with normal hypothalamic-pituitary-adrenal axis function and no hyper secretion of catecholamines. The patient was considered to have a non-secreting right adrenal mass for which an adrenal scan was done and showed a well encapsulated 10 × 9 × 7 cm heterogeneous right adrenal mass with areas of necrosis and calcifications without local invasion (Fig. 1). The decision of right laparoscopic *trans* peritoneal adrenalectomy was taken with the patient. The surgery was done under general anesthesia after central and arterial lines insertion, the patient was on left decubitus position. 5 trocars were inserted as follows: The first 12-mm port was inserted at the lateral border of the rectus abdominis muscle just above the level of the umbilicus to accommodate the camera. Two subcostal 11 mm ports were also placed; one in the midclavicular line and the other in the lateral border of the rectus abdominis muscle. The forth 5-mm subcostal trocar was inserted in the anterior axillary line to retract the liver and the fifth 5 mm one was inserted in the epigastrium and used specially for aspiration and irrigation. After liver retraction, the peritoneum along the lateral aspect of the IVC was incised to expose the IVC just below its intrahepatic course. The duodenum which was diverted by the mass was mobilized. Dissection was next carried inferiorly by incising the peritoneum along the lateral edge of the vena cava to the superior edge of the renal vein. Dissection of the mass was subsequently carried out with special care at the medial aspect where we found that the wall of the IVC and the renal vein were very adherent to the mass which had a lot of small vessels that were oozing during all the time of the surgery (Fig. 2). In addition, the mass was extended posterior to the vena cava and we could not do a medial retraction of the IVC since the tumor was adherent to it. In front of these facts, we decided to convert to open surgery by a sub-costal incision (between two trocars) that allowed us to remove the mass safely (Fig. 3). A drain was put in the retro peritoneum at the end of the surgery, the operative time was 200 min, the blood loss was 850 cc and no transfusion was done. The drain was removed at the second post-operative day and the patient was discharged uneventfully on the sixth day after surgery.

**Fig. 4:**
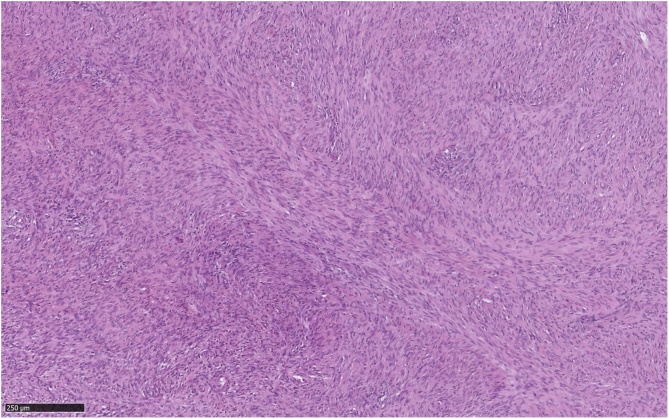
Histological aspect of the tumor: spindle cells with alternating areas of compact hypercellularity with irregular streams and without atypia or mitosis.

**Fig. 5:**
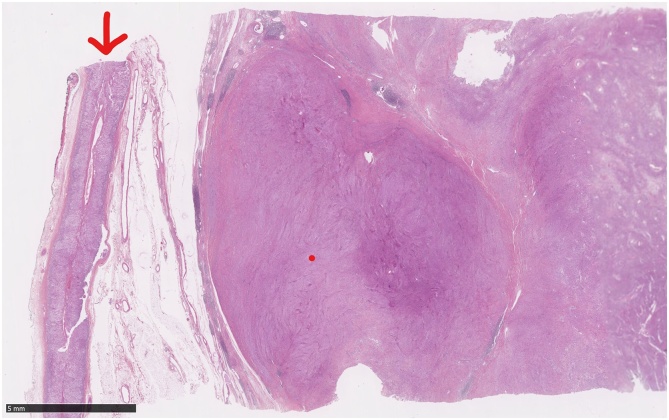
The tumor compressing and reducing the adrenal gland (red arrow) which is laminated but intact without histological abnormalities.

**Fig. 6:**
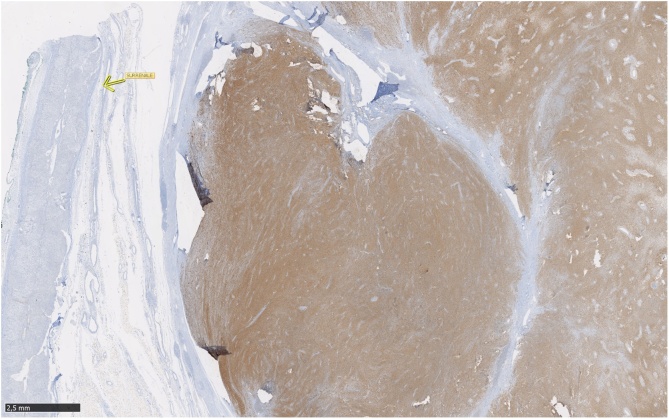
Immunohistochemical analysis: negative CKAE1-AE3, synaptophysine and chromogranine, but diffusely positive S-100 and CD68 (PGM1) staining across the tumor.

**Fig. 7:**
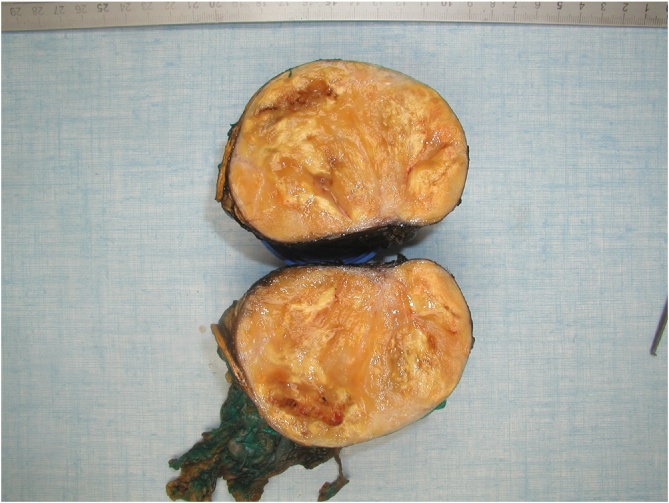
Macroscopic aspect of the benign schwannoma after cutting the tumor.

Histologically, the tumor consisted of spindle cells with alternating areas of compact hypercellularity with irregular streams and without atypia or mitosis (Fig. 4). This tumor was completely compressing and reducing the adrenal gland that was laminated but intact without histological abnormalities (Fig. 5). Immunohistochemical analysis demonstrated negative CKAE1-AE3, synaptophysine and chromogranine. In contrast to these results, S-100 and CD68 (PGM1) staining were diffusely positive across the tumor (Fig. 6). Thus, the evidences corresponded to a benign schwannoma (Fig. 7).

## 3 Discussion

Retroperitoneal schwannomas are extremely rare tumors and form 1–3% of all schwannomas that arise from Schwan cells of the peripheral nerve sheath and are usually present in cranial and peripheral nerves in the head and neck or in the upper extremities but they may appear in the posterior mediastinum, and more rarely in the retro peritoneum where they have a greater tendency to undergo spontaneous degeneration and hemorrhage when compared with other locations [7,8]. Most of schwannomas are benign tumors [9] as in our case; however, malignant schwannomas may be associated with von Recklinghausen disease [10].

The majority of these tumors is located in the paravertebral space or presacral region [7,11] and cause few symptoms at their early stage since they have a lot of space in the wide expandable retro peritoneum where they can attain massive dimensions before producing the symptoms leading to a delayed diagnosis [3]. Some of the reported symptoms are abdominal pain and distention, secondary hypertension, hematuria, and renal colic, depending on the location of the lesions. Neurologic symptoms are rare in such cases [7]. In our case, the mass was discovered late due to the absence of symptoms, the treatment was delayed which made the surgery much more complicated and difficult because of the large size of the tumor.

Retroperitoneal schwannomas usually affect patients aged 20–50 years and women are affected twice as often as men [8,12]. This goes perfectly with our patient who is a 50 Y.O female.

The preoperative diagnosis of retroperitoneal schwannomas is difficult. If they present in the suprarenal region, they may be confused with other adrenal mass lesions, especially that some case reports highlight uptake of metaiodobenzylguanidine by these lesions [4], so the definitive diagnosis is established by postoperative pathology. In our case, there was no way to know that the mass is not an adrenal tumor before the surgery and the final pathology, since it is located in the adrenal area and comprising the adrenal gland.

Imaging has certain diagnostic value but lack specificity. Liu et al. [13] reported in their case series that hypointense signal capsules in T2-weighted images in MRI and delayed enhancement of the tumor in triple phase contrast-enhanced computed tomography can be a useful way to diagnose juxta-adrenal schwannomas before the intervention. In our case, the mass was so large and it occupied the space of the adrenal gland and closely adhered to the adjacent vessels and organs which made the origin of the mass very difficult to diagnose by CT scan or MRI. Even during the operation, we thought that it was an adrenal tumor.

Macroscopically, schwannomas are a well-encapsulated solid tumor linked to a nerve. Histologically, there are three types of schwannoma: ancient, plexiform and cellular which is the most important type because it can be confused with other spindle cell malignancies. Its most important diagnosis criteria are the spindle cells with eosinophilic cytoplasm in a collagenous compact stromal with Antoni A areas (hypercellular areas) and Antoni B areas (hypocellular area). Verocay’s bodies (nuclear-free zones within the regions of nuclear palisading) can also be found. Other features suggestive of schwannoma are the presence of hyalinised vessels and infiltration of macrophages [14,15]

Immunohistochemical studies show strong positive S-100 protein (specific marker to Schwann cells and melanocytes). In our case the expression of S-100 was important to differentiate the benign nerve sheath tumor from the adrenal tissue. Negative expression of CD 34 is also valuable for the diagnosis [16]. The IHC was very useful in our case; in addition to the confirmation of the diagnosis, it detected the limits between the compressed adrenal gland and the schwannoma (Fig. 6).

Total resection is the recommended treatment of schwannomas since they are not sensitive to radiotherapy and chemotherapy. After complete resection, recurrence or malignant transformation is rare (5–10%) [17]. However, incomplete resection may lead to local recurrence in 10–54% of cases in the first 6 months postoperatively [18].

The surgical approach remains debatable: Hobart et al concluded that both laparoscopic and open surgery achieve similar results. The technique of choice could be the laparoscopic resection [19,20] but it remains controversial in larger schwannomas (>5 cm) [21]. Some authors believed that local excision can be considered for the sparing of the adjacent vital organs so they performed simple enucleation of the tumor with good results [22]. In our case we followed the approach of the radical resection instead of enucleation, assuming that we had to deal with an adrenal tumor of unknown pathology. The mass was so large that the right renal vessels and inferior vena cava represented deformation because of the compression by the mass. Although careful laparoscopic dissection and manipulation of the structures was carried out, it was difficult to excise the mass without increasing the risk of neighbor vessels and organ injury. Based on this, and in order to ensure the optimum treatment for our patient we converted to open surgery and complete excision with wide margins.

Benign schwannomas have good prognosis and the most frequent complication is recurrence, probably due to incomplete excision [17]. In malignant schwannomas, adjuvant chemotherapy or radiotherapy may be considered [23]. Our case is benign so follow-up was suggested and the patient is disease free 6 months after the surgery.

In conclusion, to our knowledge, few case series of juxta-adrenal schwannoma has been published so far, and few case reports can be found in the literature [13]. To this point of time, the preoperative diagnosis of juxta-adrenal schwannomas remains impossible, with the definitive diagnosis only established with postoperative IHC. This is why we suggest to consider the diagnosis in front of every big non secreting well encapsulated heterogeneous adrenal tumor and to even go for percutaneous biopsy, which will be very safe given the hypovascular nature of these lesions. Whatever the nature of the tumor is, the surgical difficulty is associated with the size of the mass and neighbor adhesion, so we think that early operation has to be considered in all cases independently from the surgical technique.

## Conflicts of interest

No conflict of interest.

## Funding source

No sponsor for this article.

## Ethical approval

This work is exempt from ethical approval in our institution because of its type.

## Consent

Written informed consent was obtained from the patient for publication of this case report and accompanying images. A copy of the written consent is available for review by the Editor-in-Chief of this journal on request.

## Author contribution

Bart, Coloby and Abdessater conceived of the presented idea and were encouraged by Gas to execute it.

Bart, Abdessater, El Mokdad and Sleiman were on the operating field while performing the surgery and all of them participated to the different steps of the surgery but BART was the main surgeon.

Sleiman, El Mokdad and Abessater chose and cropped the most important figures from the surgery’s video and pathology.

Gas, Sleiman and El Mokdad contributed to the final version of the manuscript. COLOBY supervised the work.

Abdessater took the lead in writing the manuscript when he found that this case deserve to be published.

All authors provided critical feedback and helped shape the manuscript.

The 6 authors designed the model and the computational framework and analyzed the results.

All persons who meet authorship criteria are listed as authors, and all authors certify that they have participated sufficiently in the work to take public responsibility for the content

## Registration of research studies

This is not a first-in-man study so it was not registered.

## Guarantor

Patrick Coloby and Stephane Bart are the guarantors of this work.

## Provenance and peer review

Not commissioned, externally peer reviewed.
